# Chemical and Structural Characterization of Amorphous and Crystalline Alumina Obtained by Alternative Sol–Gel Preparation Routes

**DOI:** 10.3390/ma14071761

**Published:** 2021-04-02

**Authors:** Izabela Rutkowska, Jakub Marchewka, Piotr Jeleń, Mateusz Odziomek, Mateusz Korpyś, Joanna Paczkowska, Maciej Sitarz

**Affiliations:** 1 Faculty of Material Science and Ceramics, AGH University of Science and Technology, 30-059 Krakow, Poland; jmar@agh.edu.pl (J.M.); pjelen@agh.edu.pl (P.J.); mateuszodziomek@gmail.com (M.O.); msitarz@agh.edu.pl (M.S.); 2 The Institute of Chemical Engineering, Polish Academy of Sciences, 44-100 Gliwice, Poland; matkor@iich.gliwice.pl; 3 Faculty of Chemistry, Jagiellonian University, 30-387 Kraków, Poland; lojewska@chemia.uj.edu.pl

**Keywords:** γ-Al_2_O_3_, amorphous-Al_2_O_3_, sol–gel, structural characterization

## Abstract

Aluminum oxide is one of the most commonly used materials in the industry. It is used in the field of catalysis, refractories, and optics. Despite the fact that there are many techniques available, there is still a great challenge in obtaining a material with desired and designed properties. Nevertheless, there is a great flexibility in making customized alumina materials with desired physicochemical properties synthesized by sol–gel methods. This work consists in characterizing the physicochemical properties of sol–gel synthesized aluminum oxide using different sol–gel preparation routes. Three different sols were obtained by using organic precursors and underwent thermal treatment. The structure (Middle Infrared Spectroscopy, Diffused Reflectance Infrared Spectroscopy, X-ray Diffraction, Magic Angle Spinning Nuclear Magnetic Resonance) and microstructure (Scanning Electron Microscopy with Electron Dispersive Spectroscopy) tests of the materials were carried out. The specific surface area was determined by using the Brunauer–Emmett–Teller (BET) method. Thermal analysis was performed for all the powders, in order to analyze the specific temperature of materials transformation.

## 1. Introduction

Alumina, although it had its glory years as a new material a few decades ago, is still one of the most important technical ceramics [1]. It has all-round uses, for instance in the field of catalysis [2,3,4], as catalyst supports or converters, as a transparent ceramic for high pressure sodium lamps or for lasers [5,6], in refractories or in electronics [7,8] as substrates for microelectronic computer chips.

It has extremely extensive applications stemming from the physicochemical properties of alumina. It is characterized with good thermal and chemical stability [9]. Its texture is defined with a high specific surface area (135–190 m^2^/g) [10,11], appropriate pore size distribution, pore size and surface acidity (derived from the local microstructure, phase composition, chemical composition). Moreover, the presence of acid/base centers may offer an additional source of active centers for catalysts [12]. Other important features are high melting point and chemical inertness, which enable the use of alumina at high temperatures or in aggressive environments. It may be accessed for a moderate price [13] which makes it an even more attractive material.

In the literature, there are various routes of obtaining alumina. Aluminas can be prepared by thermal decomposition of aluminum chemical compounds or salts or extracted from minerals. Currently, the most common route is thermal decomposition of aluminum hydroxides and oxyhydroxides [14]. The synthesis of pure alumina materials via organic or inorganic sol–gel routes has been extensively studied over recent decades due to the great technological importance of the materials and the great flexibility of the low-temperature synthetic techniques [15]. Despite the fact that there are many techniques available, there is still a great challenge in obtaining a material with desired and designed properties. Nevertheless, there is a great flexibility in making bespoke alumina materials synthesized by sol–gel method.

First and foremost, sol–gel method is one of the most well-recognized, cost competitive, bottom-up synthesis techniques [16]. Its greatest advantage is the ability to synthesize the ceramic materials from organic precursors in low temperatures. Furthermore, the method is widely used for the synthesis of crystalline and amorphous oxide materials. Materials, which are synthesized by sol–gel, may be applied for future function in different forms, for instance as powders, coatings, bulk materials, etc., depending on the technique used to fabricate them. Since the technique enables the use of a huge variety of routes of synthesis, using different types of precursors (both organic and inorganic), it is still being enhanced and refined. Undoubtfully, its development provides a great contribution in the scientific world [17].

The sol–gel method consists in preparing colloidal solutions (sols) through the hydrolysis and condensation of metal oxide precursors. The advanced condensation process, most often combined with solvent evaporation, leads to gels from which ceramic material can be obtained after thermal treatment.

The mechanism of the synthesis via sol–gel process generally requires three steps and depends on an aqueous/organic phase. It is described as follows:

1. Hydrolysis of precursors and condensation followed by polycondensation (i.e., formation of sol),
  M(H_2_O)_n_^z+^ + H_2_O → M(OH)(H_2_O)_n_^(z − 1)+^ + H_3_O^+^ (aqueous phase) (1)–M–OR + H_2_O → –M–OH + ROH (organic phase)
2. Condensation:
  2[M(OH)(H_2_O)_n_^(z − 1)+^] → [M–O–M(OH)_2_(H_2_O)_2n − 1_]^2(z − 1)+^ + H_2_O (aqueous phase)
–M–OH + RO–M → –M–O–M– + ROH or –M–OH + HO–M → –M–O–M– + H_2_O (organic phase)
  where M refers to metal atom, R to alkyl groups and n to metal valency. The rate of these reactions can be controlled by changing pH. In addition, parameters such as temperature, humidity, stirring speed, solubility of the reagents and other factors may have impact on the process [18].

During condensation, the three-dimensional structure of the gel undergoes the formation of a sol. As the polymerization progresses, the particle weight and viscosity of the sol increases, resulting in coagulation of colloidal particles.

3. Gelation, known as sol–gel transformation, which leads to the formation of a jelly like substance—a gel of high softness.

There are many parameters which significantly affect the course and reproducibility of the synthesis, parameters which determine the chemical composition, structure and morphology of material obtained by sol–gel method [19]. The most important one is the hydrolysis ratio. It demines the structure of the product, viscosity and the condensation/gelation time. The lower amount of water added results in increasing viscosity of the sol and low crosslinking product, while a higher amount had the opposite effect [20]. The chemical composition of the precursor affects the final product and the reaction kinetics, the presence of acid or base catalyst increase the rate of polymerization. pH has an impact on the particle size of the sol, while the temperature results in both hydrolysis and condensation rate increase [21].

Aluminum hydroxides and oxo-hydroxides are commonly used precursors to transition and amorphous aluminas. Synthesis can be carried out in both aqueous, inorganic solutions and non-aqueous solution derived from metal alkoxides [22,23].

Aluminum hydroxide derived from the hydrolysis of aluminum alkoxide gives bayerite or gibbsite structures when the reaction temperature is below 80 °C and otherwise boehmite structure [24].

Therefore, different approaches to sol–gel synthesis have been systematized. The first standardized type, which was developed by B.E. Yoldas [25,26,27], involves hydrolysis of aluminum alkoxides in excess water and subsequent peptization and acidification in acid solutions. Alternate procedure, which utilizes chelating agents and aims at the control of the hydrolysis and condensation rates, also shows great flexibility in tailor-making alumina materials with desired physicochemical properties [28]. A chelating agent, i.e., acetylacetone, are chosen in order to reduce the complexity of the reactions and therefore they control the rates of hydrolysis and condensation and also determine the final properties of the alumina materials. Since the sol–gel method is still under development, there are many improved ways of obtaining materials in using such routes.

The principal features of the Al_2_O_3_ microstructure are usually reported for the oxide obtained by the thermal dehydration (calcination) of aluminum hydroxides and oxyhydroxides [29]. Therefore, the properties of the expected alumina depend on the method and form it is derived from. Appropriately selected parameters significantly affect the characteristics of the final material obtained by the sol–gel method.

Alumina during thermal treatment occurs in different phase transformations. The stoichiometric formula of Al_2_O_3_ of various phases is described in the following sequence, which can be obtained by varying the calcination temperatures of aluminum hydroxide and aluminum oxyhydroxide [30]:

Al(OH)_3_ → AlOOH → a–Al_2_O_3_ → γ-Al_2_O_3_ → δ-Al_2_O_3_ → θ–Al_2_O_3_ → α–Al_2_O_3_

In this paper, we aim to characterize the properties of alumina powders before heat treatment and after, obtaining γ- and amorphous-alumina. For that, we synthesized three different aluminas via the sol–gel process, using alternative routes. Although similar syntheses have already been described, the obtained products have never been fully characterized. Therefore, detailed structure (Middle Infrared Spectroscopy, Diffused Reflectance Infrared Spectroscopy, X-ray Diffraction, Magic Angle Spinning Nuclear Magnetic Resonance) and microstructure (Scanning Electron Microscopy with Electron Dispersive Spectroscopy) analysis has been provided. The specific surface area was determined by using the Brunauer–Emmett–Teller (BET) method. Thermal analysis was performed for all the powders, in order to analyze the specific temperature of alumina transformation.

## 2. Materials and Methods

### 2.1. Sols Preparation

The first sol, S1, was prepared using B. E. Yoldas formula [25,26,27]. Aluminum tri-sec-butoxide (Al(O-sec-Bu)_3_, ASB) with butan-2-ol, was hydrolyzed in water. Then, the mixture was heated to 80–90 °C for 30 min with stirring. Nitric acid (HNO_3_) was added to the mixture after it was cooled down to room temperature, as acidification agent. Two following alumina sols, S2 and S3, were prepared by hydrolysis and polycondensation of ASB in propan-2-ol (for S2) or in butan-2-ol (for S3) as solvent with 2,4-pentanedione (acetylacetone) as chelating agent and hydrochloric acid (HCl) as acidification agent.

### 2.2. Powder Preparation

Synthesized sols were poured out to dry in the air for 24 h, at room temperature. Powders were put into the furnace and fired in 600 °C, ramping 10 °C/min, held S1 for 30 min, S2 and S3 for 60 min The temperature was established due to the results of thermal analysis.

### 2.3. SEM

Scanning Electron Microscopy (SEM) and Electron Dispersive Spectroscopy (EDS) was performed with use of Phenom XL (Thermo Fisher Scientific, Waltham, MA, USA). Secondary electrons detector was used with the 15 kV acceleration voltage applied while analysis. The results of EDS were provided as average analysis from the area of 1 mm × 1 mm.

### 2.4. XRD

High-temperature X-ray diffraction measurements were carried out in the range of 20°–70° using CuKα radiation on Empyrean (Malvern Panalytical, Malvern, UK) diffractometer, equipped with PIXcel3D detector (Malvern Panalytical, Malvern, UK). For high temperature studies, HTK 1200N (Anton Paar, Graz, Austria) oven-chamber was used. Data collection was performed at a temperature range of 20 to 600 °C, measurement every 10 °C. Before the measurement, the sample was equilibrated at a particular temperature for 30 s.

### 2.5. FTIR and DRIFT

Middle infrared spectroscopy—for Fourier-transformed infrared (FTIR) examination, a Vertex 70 v (Bruker, Billerica, MA, USA) spectrometer was used. Spectra were recorded using standard KBr pellet method as well as in situ DRIFTS (Diffused Reflectance Infrared Spectroscopy) in the 400–4000 cm^−1^ range with 4 cm^−1^ of resolution and 128 scans. Spectral deconvolution was carried out using Bruker OPUS software (version 7.2, FT-IR Spectroscopy Software, Bruker, Billerica, MA, USA). Levenberg–Marquart iteration algorithm was employed. A set of Gaussian–Lorentzian functions was used with the starting ratio equal to 0.5. For each sample, RMS error at the end of the procedure was recorded to be below 0.1.

### 2.6. NMR

Magic Angle Spinning Nuclear Magnetic Resonance (MAS NMR) spectra were recorded on the Apollo (Tecmag, Houston, TX, USA) spectrometer console. A Magnex superconducting magnet with a gap at the level of 89 mm producing a magnetic field equal to 7 T was used. An HP WB 73A (Bruker, Billerica, MA, USA) CP-MAS probe was equipped with a zirconia rotor with a diameter of 4 mm, where powder samples were placed and spun with a KEL-F turbine using the magic angle at a 4–8 kHz frequency. The frequency scales [ppm] ^27^Al was determined with aluminum nitrate (Al(NO_3_)_3_).

### 2.7. BET

The specific surface area (SSA) was evaluated from BET adsorption isotherms (Brunauer–Emmett–Teller) on the ASAP 2010 unit (Micromeritics Instrument Corporation, Norcross, GA, USA). The measurement was conducted with the N_2_ usage (99.999%, Air Liquide, Kraków, Poland).

## 3. Results and Discussion

### 3.1. SEM and EDX Analysis

The microstructure and chemical composition of obtained samples, before and after heat treatment were characterized by scanning electron microscopy coupled with EDS. The results of the EDS microanalysis are presented in the Table 1.

The elemental composition, based on the EDS examination of prepared materials, is very similar/identical for all samples. Samples consist of two elements: aluminum and oxide. Samples before heat treatment suggest that the synthesis product for S1 can be boehmite (AlOOH) and for the samples S2 and S3 it can be aluminum hydroxide (Al(OH)_3_). For all the samples after the heat treatment, the ratio is equivalent to aluminum oxide. The atomic ratio between aluminum and oxygen Al:O is for the samples before heat treatment 1:2, 1:3, 1:3, respectively. It confirms that the product of the synthesis may be AlO(OH) and Al(OH)_3_. For the samples after heat treatment all the ratios have the value 2:3, which stands for Al_2_O_3_. The calculation of the atomic ratios of the elements, however, cannot be the only basis for drawing structural conclusions. Therefore, further structural examination was performed.

**Figure 1:**
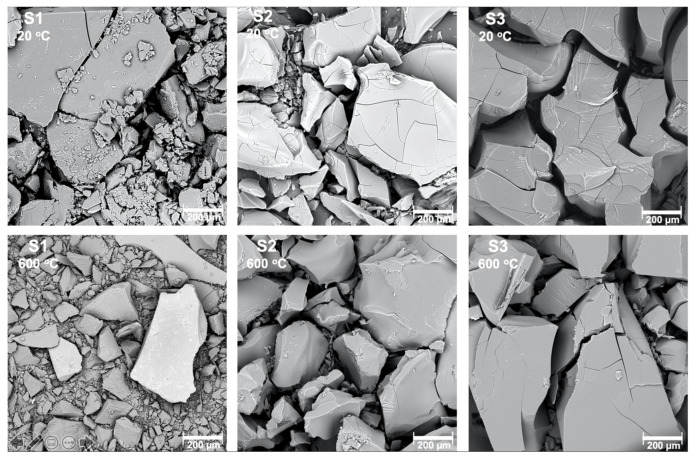
Scanning electron microscope pictures of the samples S1, S2, S3 before and after heat treatment in 600 °C.

All the samples were obtained as fine powders (Figure 1). After heat treatment, the microstructure of the samples had not changed, however transparency was gained by all of them.

### 3.2. FTIR

**Figure 2:**
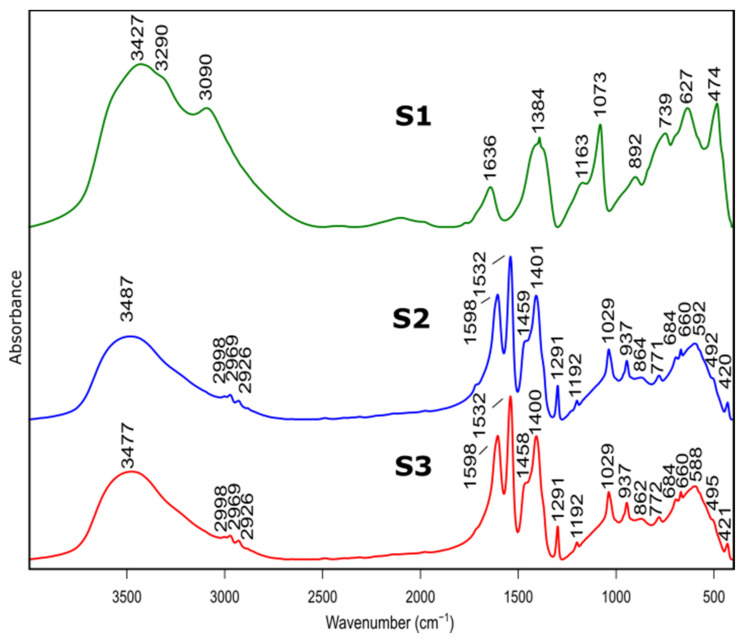
FTIR spectra of as prepared samples S1, S2, S3 before heat treatment.

**Figure 3:**
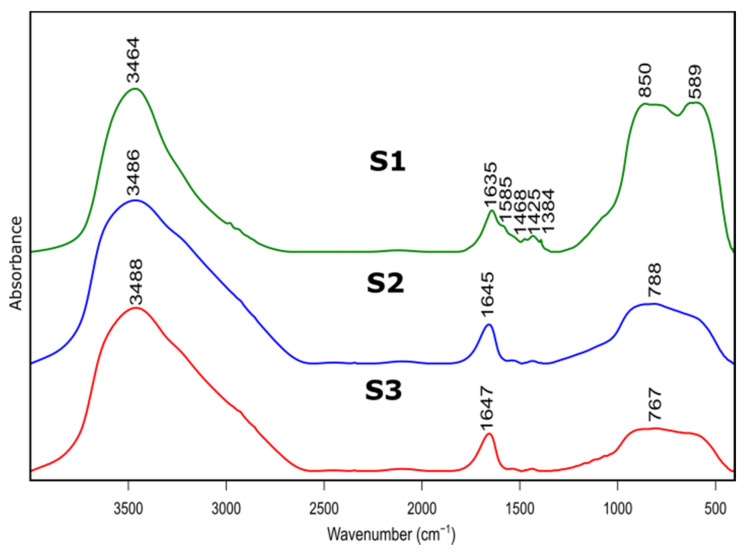
FTIR spectra of as prepared samples S1, S2, S3 after thermal treatment at 600 °C.

The samples before and after calcination were investigated by FTIR. The spectra are shown in Figure 2 and Figure 3 and the peak assignment in Table 2 [31,32].

Bands at 3427 cm^−1^, 3481 cm^−1^ and 3474 cm^−1^ belonged to S1, S2, S3 spectra, respectively, and are assigned to stretching vibration of –OH group present in physically absorbed water. This is confirmed by the band at 1636 cm^−1^, for S1 spectrum, originating from bending–scissoring vibrations (H–O–H) typical for water molecules. In addition, bands at 3090 cm^−1^ and 3290 cm^−1^, visible in the S1 spectrum, are typical for AlOOH structure and originate from stretching vibrations of –OH. Bands present at 1163 cm^−1^ and 1073 cm^−1^ correspond to in-plane bending–scissoring vibration of OH in Al–O–H. Bands observed at 739 cm^−1^, 627 cm^−1^ and 474 cm^−1^ are assigned to stretching and bending–scissoring vibrations between aluminum and oxide in octahedral aluminum [AlO_6_]. The Al–O stretching vibrations in [AlO_4_] tetrahedral configuration (S2 and S3 spectra) are visible in the range of 862 to 592 cm^−1^. Due to the usage of nitric acid as a catalyst during synthesis of S1 sample vibrations typical for nitrate compounds are also visible—1384 cm^−1^ and 892 cm^−1^.

For the S2 and S3 samples, bands observed in the range of 2998 to 2926 cm^−1^ come from the C–H stretching vibrations in the Al(acac)_3_ (aluminum acetonate) formed while the chelating agent is added. Bands at ranges 1598–1532 cm^−1^ and 1459–1400 cm^−1^ are assigned to C=O stretching vibration and C-H bending vibrations, respectively. Moreover, vibrations visible at 1192 cm^−1^, 1029 cm^−1^ (for sample S2) and 1291 cm^−1^, 937 cm^−1^ (for sample S3) derive from stretching and in-plane bending modifications between C–CH_3_ groups in Al(acac)_3_. The stretching vibrations between Al–O–C results in bands at around 492 cm^−1^ and 420 cm^−1^. The presence of these bands is due to the use of the acetylacetone during S2 and S3 synthesis as a chelating agent.

The spectra of S2 and S3 samples are very similar (e.g., band positions and intensities), indicating that despite the use of different solvents, obtained products are aluminum hydroxide (Al(OH)_3_).

Presented results confirm that the product of the synthesis of the samples S1 is boehmite (AlO(OH)) while for the samples S2 and S3, it is aluminum hydroxide (Al(OH)_3_).

**Figure 4:**
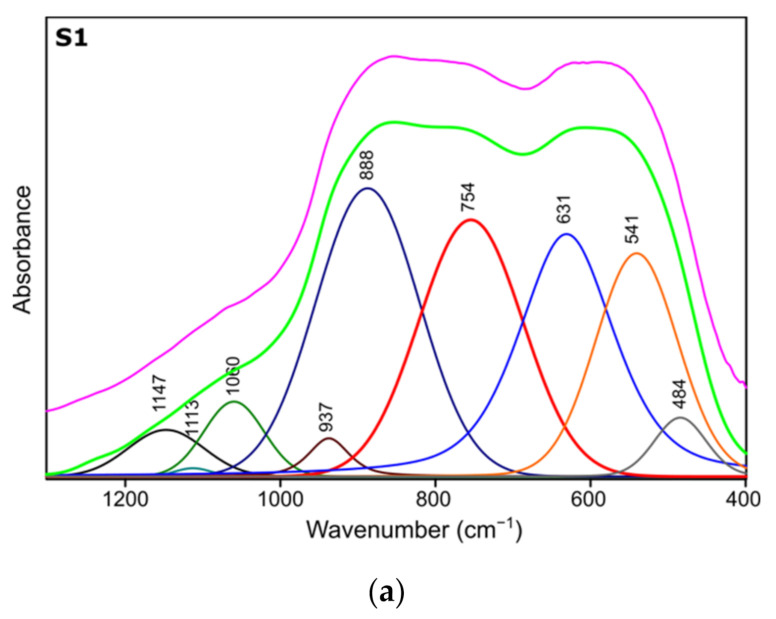

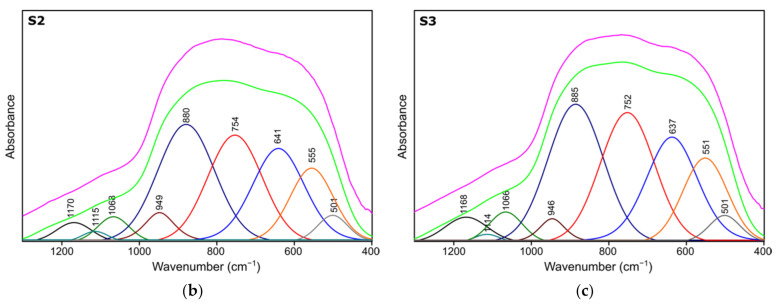
Spectra deconvolution of the samples (**a**) S1, (**b**) S2, (**c**) S3.

Figure 3 presents FTIR spectra of S1-S3 samples after thermal treatment. Positions and assignment of the bands is shown in Table 3. Bands at approximately 3480 cm^−1^ visible on all recorded specimens can be attributed to the presence of –OH species, typical for absorbed water molecules. This is confirmed by the presence of H–O–H bending band at approximately 1640 cm^−1^. Bands at approximately 1425 cm^−1^ probably correspond to carbonate traces. Bands at 850 cm^−1^, 788 cm^−1^ and 767 cm^−1^ refer to bending–scissoring vibration between Al–O–Al. The band at 589 cm^−1^, which is mainly observed for the S1 sample, is attributed to Al–O stretching mode and indicates the presence of Al in tetrahedral and octahedral configurations. Due to the complex nature of bands in the range of 400 to 1300 cm^−1^, spectral deconvolution to component bands was performed. The abovementioned process was carried out using Bruker OPUS software and final results were presented in Figure 4 and Table 4.

Results of spectra deconvolution are presented in Figure 4 and Table 4. Based on the given data, it is clearly visible that all of the tested samples exhibit same spectral features—similar band positions and integral intensities. Presented in Table 4, data suggest that after thermal treatment all of the obtained materials have γ-Al_2_O_3_ structure. This is confirmed by the presence of Al–O and Al–OH bands belonging to [AlO_4_] and [AlO_6_] species typical for gamma-alumina [32,34]. There are also visible bands typical for vibrations of OH bonds in and on the surface of Al_2_O_3_ [32,36]. There are slight differences between the individual samples in the bands positions, their integral intensities and width (Full Width and Half Maximum). The analysis of the obtained results shows that the S1 sample is the most crystalline. This is observed through the smallest half-widths of the obtained bands. The component bands of the S2 and S3 spectra are characterized by higher FWHM values, which suggests their more amorphous nature, which has been confirmed by X-ray studies. At the same time, the observed changes in the integral intensities indicate that the S1 sample contains the most Al–O bonds originating from octahedrons [AlO_6_] characteristic vibrations typical for the gamma-alumina phase [32,33].

The FTIR analysis indicate that samples after thermal treatment transform into aluminum oxide. Moreover, detailed spectroscopic studies confirmed that S1 sample is γ-Al_2_O_3_ while S2, S3 samples correspond to a more amorphous- γ-Al_2_O_3_-like structure. Presented results correspond with both XRD and NMR analysis.

### 3.3. DRIFTS

**Figure 5:**
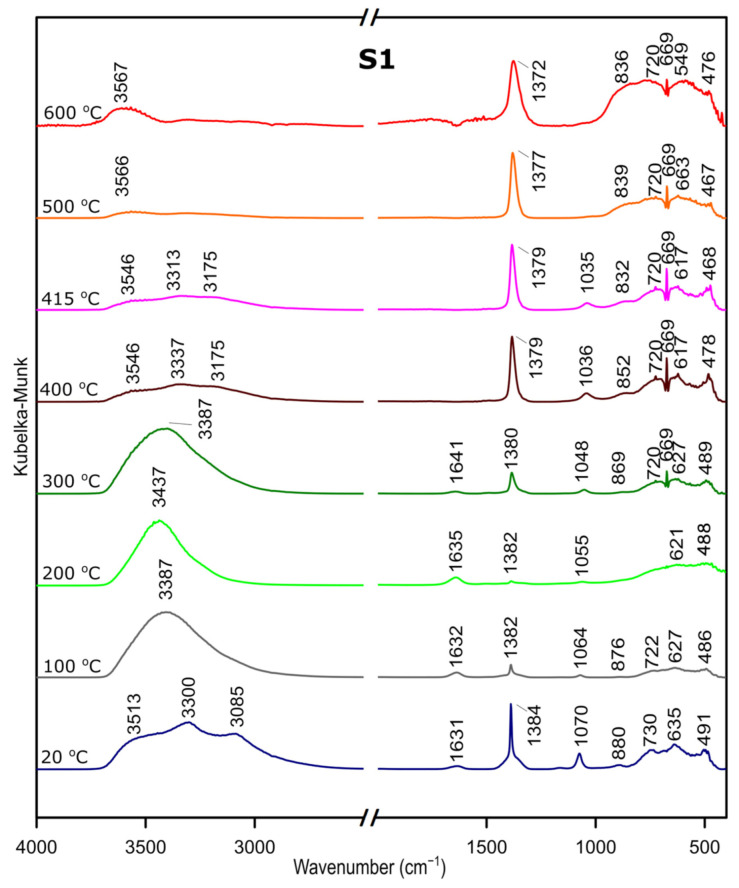
DRIFTS results for the sample S1.

**Figure 6:**
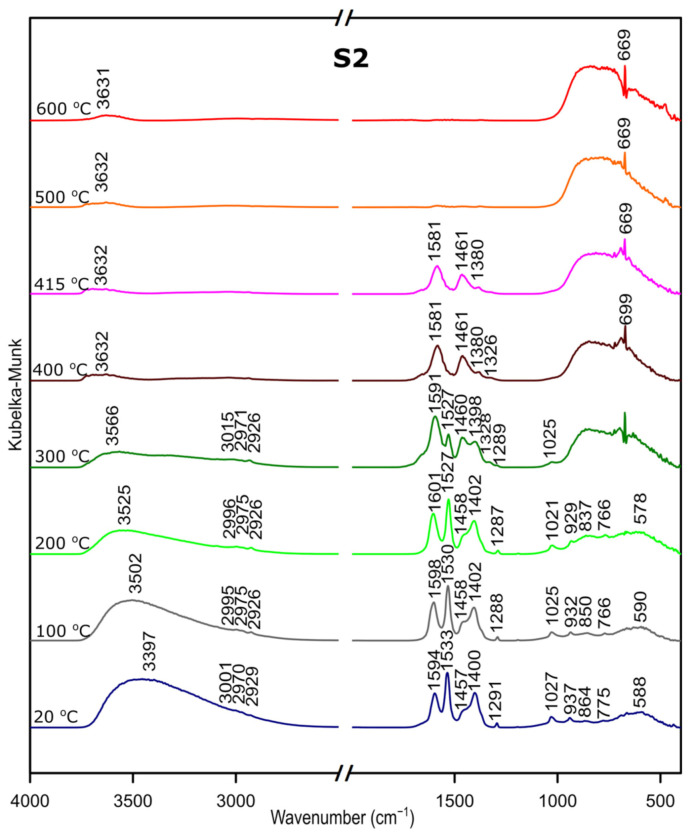
DRIFTS results for the sample S2.

**Figure 7:**
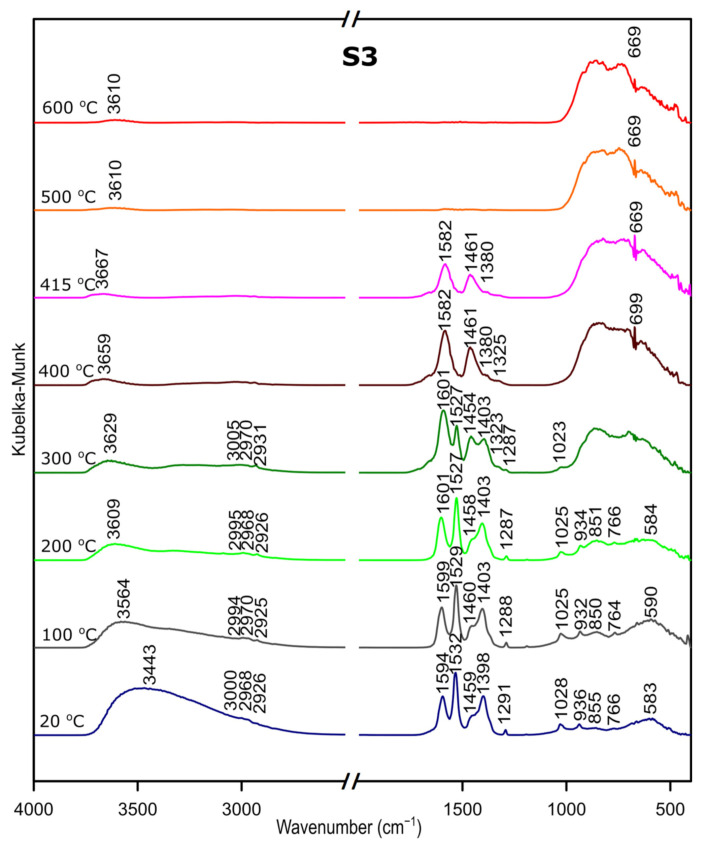
DRIFTS results for the sample S3.

We followed the transformation of alumina gel into alumina oxide during calcination process by in situ FTIR. The diffusive reflectance was used because it enables the observation of the structural changes in temperature function. Figure 5, Figure 6 and Figure 7 present the results of DRIFTS for the samples S1, S2, S3, respectively. In order to present the results clearly, the range 2000–2500 cm^−1^ has been omitted. Bands, which were present in the range mentioned, come from carbon dioxide released during the heating process and their interpretation is not relevant to the analysis. The 669 cm^−1^ band located on the spectra for all samples in the range from about 300 to 600 °C also comes from the emission of carbon dioxide during combustion of organic precursors and solutions used during syntheses.

In the zone between 3700 and 2900 cm^−1^, corresponding to stretching vibration of molecular water and hydroxyl groups, bands at 20 °C are attributed, respectively, to the asymmetrical and symmetrical stretching vibrations of bulk hydroxyls and at about 1640 cm^−1^, to the band corresponding to the H–O–H bending–scissoring vibration from physiosorbed water. With the temperature increase, there is an elimination of traces of physiosorbed water from the surface and the band at around 1630 cm^−1^ disappears. Similarly, there is a shift of the bands in the 4000–2800 cm^−1^ range and bands above 3500 cm^−1^ and over the temperature of about 500 °C can be attributed to stretching modes of the terminal –OH on the Al ions. The bands at lower wavenumbers, at 1370–1385 cm^−1^, are probably due to nitrate species present on the surface [37]. Nitric acid was added during preparation to adjust the pH of the solutions.

The band at around 1070–1035 cm^−1^ corresponds to OH bending–scissoring vibration of Al–O–H in AlOOH and disappears above around 425 °C.

Bands in the range from 730 to 450 cm^−1^ are assigned to Al–O stretching and bending–scissoring vibrations in octahedral and tetrahedral aluminum. It is observed that with the increase in the temperature, above about 425 °C the intensity of the bands is increasing, which causes the γ-aluminum oxide formation [38].

All observations and DRIFTS analyses enable us to state the following conclusion: with the temperature increase, around 425 °C, there is a transformation from boehmite to the γ-Al_2_O_3_. Moreover, the conversion is observed at the similar temperature as thermal analysis studies, therefore we can conclude that there is a great agreement between the Differential Scanning Calorimetry and DRIFTS analyses concerning the temperatures observed relative to the departure of water and the conversion to the γ-Al_2_O_3_.

Considering the fact that the analyzed starting materials are the same, interpretation was performed jointly for both S2 and S3 samples, spectra S2 and S3 (Figure 2 and Figure 3).

In the OH stretching zone between 3700 and 2900 cm^−1^, bands at 20 °C are attributed, respectively, to the asymmetrical and symmetrical stretching vibrations of hydroxyl groups from water and with the temperature increase, up from about 400 °C they transform into stretching modes of the terminal –OH on the Al ions with tetrahedral coordination. Small bands in the range between 3015 and 2925 cm^−1^ correspond to C–H stretching vibration, 1600 to 1530 cm^−1^ are attributed to C=O stretching vibration and 1470 to 1280 cm^−1^ come from C–H bending vibration in Al(acac)_3_. Above 415 °C, the bands are disappearing; this proves the organic combustion.

Bands in the range from 770 to 400 cm^−1^ are assigned to stretching and bending–scissoring vibrations in aluminum and oxide in octahedral and tetrahedral aluminum. Similar to the sample S1, as the temperature increases, the intensity of the bands is increasing.

The transformation to aluminum oxide is recorded at around 420 °C, showing the good correlation between thermal analyses.

### 3.4. XRD

**Figure 8:**
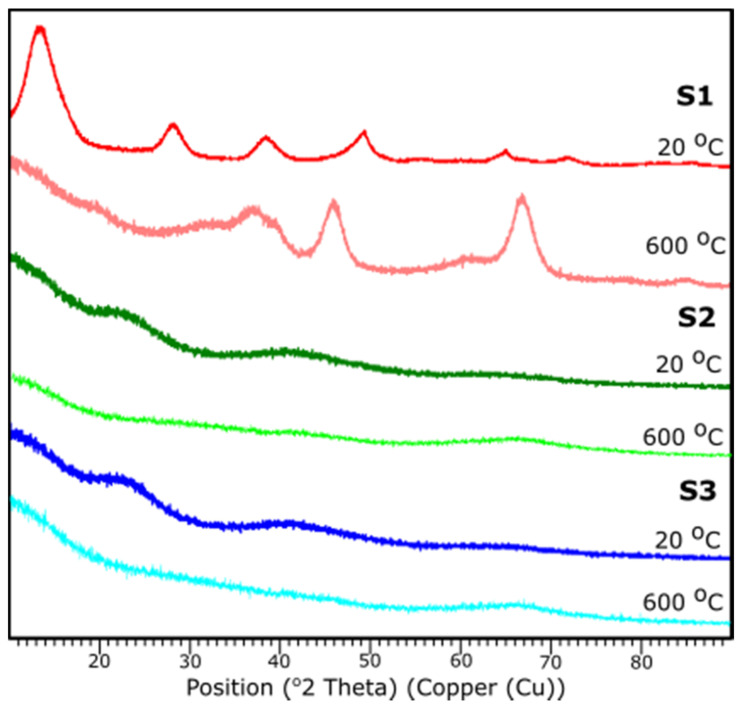
XRD diffraction patterns of the samples S1, S2, S3 at 25 and 600 °C.

Figure 8 presents the results of XRD examination of all samples before and after thermal treatment. All diffraction peaks are determined according to JCPDS database.

Based on the XRD analysis, S1 indicates the crystalline structure of the material before and after thermal treatment. According to the card No. 21-1307 database [39], at 20 °C the peaks stand for the pattern adequate to aluminum oxide hydroxide, AlO(OH). Reflections suit an orthorhombic crystal system with the space group: Cmc21. Peaks 14.48, 28.11, 38.25, 45.65, 48.81, 51.44, 55.09, 60.45, 63.88, 64.78, 67.53, 71.73, respectively, corresponds to h, k, l positions (020), (120), (031), (131), (051), (220), (151), (080), (231), (002), (171), (251).

The sample S1 after the thermal treatment represents diffraction peaks characteristic to γ-Al_2_O_3_, according to JCPDS Card No. 29-0063 database. Indicated peak list 37.60, 39.49, 45.79, 60.89, 66.76 corresponds, respectively, to the (311), (222), (400), (511), (440). Identified peaks refer to the reflections of the cubic crystal system of γ-Al_2_O_3_ with lattice constant of a = b = c = 0.790 nm, space group: *Fd3m.*

For the samples S2 and S3 no crystalline peaks are detected, thus the structures can be considered as amorphous or very fine particles. A characteristic diffraction halo is observed in the XRD pattern, suggesting that an amorphous phase of Al_2_O_3_ was formed [40]. XRD measurements correspond with FTIR results.

### 3.5. Temperature XRD

**Figure 9:**
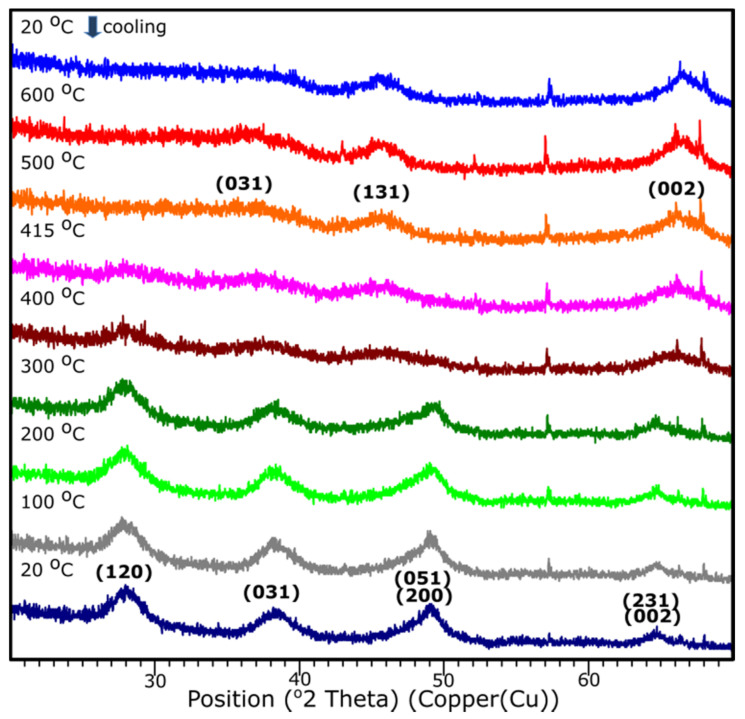
XRD diffraction patterns of the sample S1 with the temperature growth from 20 to 600 °C and subsequent cooling to 20 °C.

The temperature XRD analysis was applied in order to present the transition to the γ-Al_2_O_3_. Results are presented in Figure 9. Sample S1 underwent the examination, since based on the XRD examination, it presents the crystalline nature. It is shown that the transition from aluminum oxide hydroxide (AlO(OH)) to γ-Al_2_O_3_ occurs around 415 °C. The diffraction patterns from the temperature 25–405 °C stands for the pattern represented by the AlO(OH) (boehmite) which transforms into γ-Al_2_O_3_ with the temperature growth. The results correspond well with the DRIFTS analysis.

### 3.6. NMR

**Figure 10:**
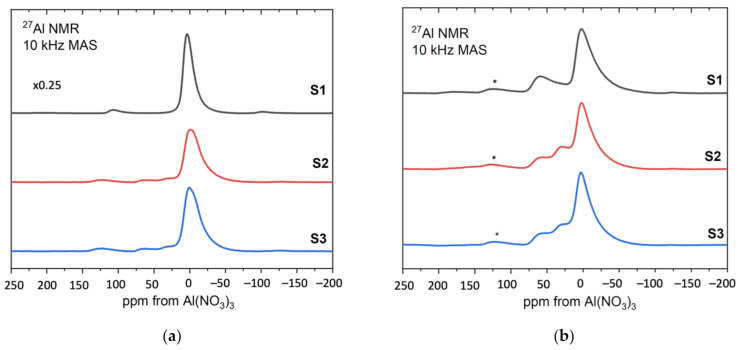
The ^27^Al MAS NMR spectra of the samples S1, S2, S3—(**a**) before and (**b**) after heat treatment.

Figure 10 shows the ^27^Al MAS NMR spectra obtained from the sol–gel produced alumina S1, S2, S3 [39,40].

Figure 10a presents the results for the samples before heat treatment. For comparison purposes, the spectrum of sample S1 was reduced to ¼. Resonance at around 0 ppm corresponds to aluminum octahedral coordination and its broadened resonance/line is present at all samples S1, S2, S3. In addition, in samples 2 and 3, noticeable component contributions can be seen at 30 ppm and 50 ppm, which are assigned to aluminum pentagonal and tetrahedral coordination, respectively. Since boehmite contains only octahedrally coordinated aluminum, the NMR results for the sample S1 confirm the boehmite as a synthesis product.

Figure 10b shows the results for S1, S2, S3 after heat treatment at 600 °C [41,42]. The relative amounts of the three different aluminum coordinations: tetrahedral, pentahedral and octahedral are visible at approximately 70, 35 and 0 ppm, respectively, for all samples. As the annealing temperature increases to 600 °C, hydroxyl groups remaining from the sol–gel formation process are removed from the system and the boehmite is converted to a transition alumina phase with significant inherent disorder as indicated by the presence of five-coordinated aluminum.

### 3.7. Thermal Analysis TG, DTG, DSC

**Figure 11:**
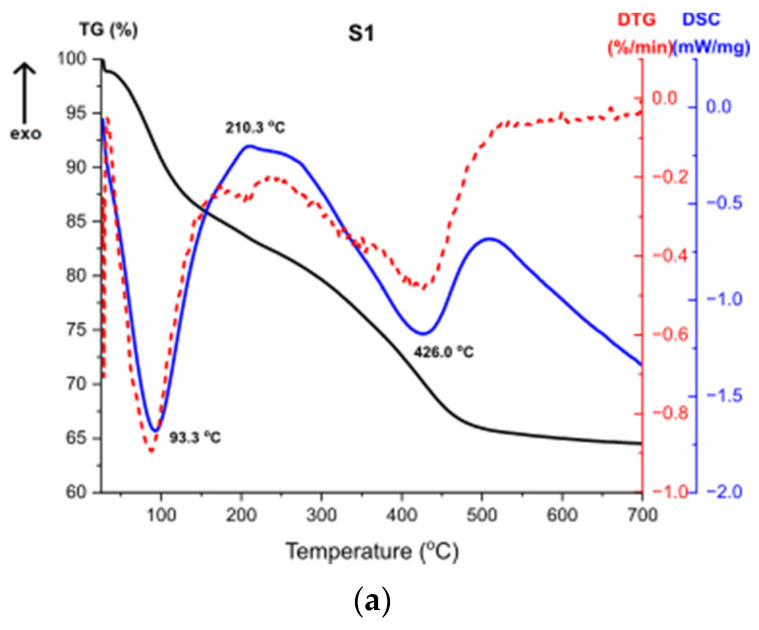

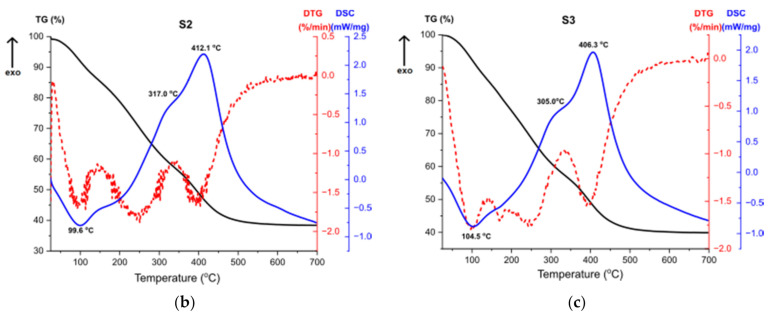
TG, DTG, DSC curves for the samples (**a**) S1, (**b**) S2, (**c**) S3.

Figure 11 presents the results of Thermogravimetry (TG), Derivative Thermogravimetry (DTG) and Differential Scanning Calorimetry (DSC) [43,44]. Figure 11a shows the curves for the sample S1. The first endothermic peak visible on the DSC curve at around 93 °C (Δm1 = −15.52%) is caused by the partial dehydroxylation of molecular water and solvent evaporation, which is indicated by the first weight loss recorded on the TG/DTG curve. The exothermic peak at around 210 °C corresponds to the combustion of organics formed during the synthesis. It is accompanied by the weight loss on the TG curve Δm2 = 2.07%. Another endothermic peak at 426 °C is caused by the decomposition of boehmite and the formation of γ-Al_2_O_3_ aluminum oxide. Continuous measurement during thermal analysis enables the determination of the exact temperature of the transformation. Moreover, there is a structural water evaporation, reflected in another weight loss (Δm3 = −17.87%). The total mass loss is around 35%.

Thermal analysis results for the sample S3, shown at Figure 11c, are almost the same as for the sample S2 (Figure 11b), which proves that the result of the synthesis is the same product and additionally confirms the results of the structural analysis. The first endothermic peak refers to solvent evaporation, with two accompanying weight losses. High exothermic effect at 305 °C corresponds to the combustion of organics [45]. DTG curve shows a multi-stage weight loss associated with this effect, presented by two weight losses. The second exothermic peak at 406 °C stands for the transformation of the amorphous alumina from the breakdown of Al(OH)_3_ and the structural water evaporation [46].

The thermal analysis revealed that samples S2 and S3 after the synthesis were composed with bigger number of organic compounds compared to the sample S1. Therefore, during heating, greater weight loss for the given samples is observed.

### 3.8. BET Examination

**Figure 12:**
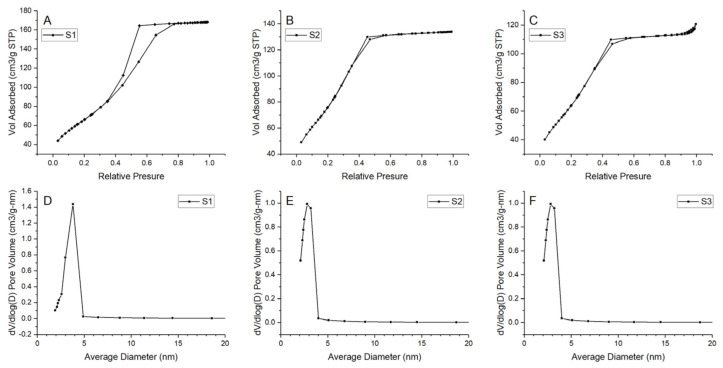
N_2_ adsorption–desorption isotherms and pore size distribution for the samples (**A**), (**D**) S1, (**B**), (**E**) S2, (**C**), (**F**) S3.

Prepared N_2_ adsorption–desorption isotherms and pore-size distributions for all samples are presented in Figure 12. The results indicate formation of a mesoporous structure in all samples. For considered samples, type IV isotherm can be matched with a hysteresis loop in the relative pressure range of 0.4–0.8, indicating the generation of mesopores in the samples [47]. Pore size distribution is uniform with one main peak in the ranges of 2–5 nm. However, for S1, the range seems to be wider than for the samples S2 and S3. Additionally, samples S2 and S3 have shifted the distribution maximum to the smaller value. Thus, it should be noted that there might also be a small number of micropores in samples S2 and S3 because the dV/d(log(D)) value decreased with the increase in pore size in the range of 2–5 nm. Detailed information about the BET surface area and pore value are presented in Table 5. BET surface area differs among samples—for samples S2 and S3 it equals 274.524 and 271.328 m^2^/g, respectively, and is much higher than those of the S1 (238.395 m^2^/g). The largest pore volume is exhibited by sample S1 (0.259 cm^3^/g).

## 4. Conclusions

The aim of the research was a structural and chemical characterization of aluminum oxide obtained by using two different routes of sol–gel synthesis technique.

The structural studies confirmed obtaining two different aluminum oxide forms: S1 γ-Al_2_O_3_ and S2 and S3 amorphous γ-Al_2_O_3_-like structure. FTIR, NMR and XRD correlation enable us to state that abovementioned structures were obtained. The DRIFTS and thermal analysis correlation allowed us to estimate the approximate transformation temperature for all samples S1, S2, S3 which are around 425 °C, 415 °C, 415 °C, respectively. SEM combined with EDX confirmed that the obtained materials after heat treatment present aluminum oxide.

The BET studies correspond to the structural studies, showing the different degrees porosity and surface area for obtained materials. All samples are characterized with the surface area higher than 230 m^2^/g.

Considering the examined properties of the obtained materials, future application as thin layers which act as catalyst carriers may be possible.

## Figures and Tables

**Table 1:** Electron Dispersive Spectroscopy (EDS) microanalysis.

S1 20 °C	Al	O	S1 600 °C	Al	O
At%	33.07	66.93	At%	38.16	61.84
Wt%	45.45	54.55	Wt%	50.99	49.01
S2 20 °C	Al	O	S2 600 °C	Al	O
At%	26.42	73.58	At%	36.62	63.38
Wt%	37.72	62.28	Wt%	49.35	50.65
S3 20 °C	Al	O	S3 600 °C	Al	O
At%	26.28	73.72	At%	38.07	61.93
Wt%	37.55	62.45	Wt%	50.90	49.10

**Table 2:** Positions and assignments of the bands from FTIR reflectance spectra of samples S1, S2, S3 in room temperature.

S1 (cm^−1^)	S2 (cm^−1^)	S3 (cm^−1^)	Type of Vibration
3427 s, br	3481 s, br	3474 s, br	ν_as_ and ν_s_ O-H in H_2_O
3290 s, sh	-	-	ν_as_ OH in AlOOH
3090 s	-	-	ν_s_ OH in AlOOH
-	2998 w	2998 w	ν C-H in Al(acac)_3_
-	2969 w	2969 w	ν C-H in Al(acac)_3_
-	2926 w	2926 w	ν C-H in Al(acac)_3_
1636 m			δ_s_ H-O-H in H_2_O
-	1598 s	1598 s	ν C = O in Al(acac)_3_
-	1532 vs	1532 vs	ν C = O in Al(acac)_3_
-	1459 m, sh	1458 m, sh	δ C-H in Al(acac)_3_
-	1401 s	1400 s	δ C-H in Al(acac)_3_
1384 m	-	-	ν_as_ N-O in NO_3_^−^
-	1291 m	1291	ν C-C in Al(acac)_3_
-	1192	1192	δ C-CH_3_ in Al(acac)_3_
1163 m, sh	-	-	δ_as_ OH of Al-O-H in AlOOH
1073 m	-	-	δ_s_ OH of Al-O-H in AlOOH
-	1029 m	1029 m	δ C-CH_3_ in Al (acac)_3_
-	937 m	937 m	ν C-CH_3_ in Al(acac)_3_
892 m	-	-	ν_s_ N-O in NO_3_^−^
-	864 w	862 w	ν Al-O of [AlO_4_] in Al_2_O_3_
-	771 w	772 w	ν Al-O of [AlO_4_] in Al_2_O_3_
739 m	-	-	ν Al-O of [AlO_6_] in AlOOH
-	684 w	684 w	ν Al-O of [AlO_6_] in Al_2_O_3_
-	660 w	660 w	ν Al-O of [AlO_6_] in Al_2_O_3_
627 s	-	-	ν Al-O of [AlO_6_] in AlOOH
-	592 m	588 m	ν Al-O of [AlO_6_] in Al_2_O_3_
-	492 w, sh	495 w, sh	ν Al-O-C in Al(acac)_3_
474 s	-	-	δ Al-O of [AlO_6_] in AlOOH
-	420 w	421 w	δ Al-O-C in Al(acac)_3_

**Table 3:** Positions and assignments of the bands from FTIR reflectance spectra of samples S1, S2, S3 in 600 °C.

S1 (cm^−1^)	S2 (cm^−1^)	S3 (cm^−1^)	Type of Vibration
3464 s, br	3486 s, br	3488 s, br	ν_as_ and ν_s_ O-H in H_2_O
1635 m	1645 m	1647 m	δ_s_ H-O-H in H_2_O
1468 m	-	-	ν_as_ N-O in NO_3_^−^
850 w	-	-	δ_s_ or ν Al-O of [AlO_4_] in Al_2_O_3_ combined bands
-	788 w	767 w	ν Al-O of [AlO_4_] in Al_2_O_3_ combined bands
589 w	-	-	ν Al-O of [AlO_6_] in Al_2_O_3_ octahedral and tetrahedral

**Table 4:** Positions and assignments of the bands from spectra deconvolution for samples S1, S2, S3.

Sample	Band Assignment	ν_as_ Al-O [AlO_6_]/ δ Al-O [33,34]	ν Al-O of [AlO_6_] [35]	ν_s_ Al-O of [AlO_6_] [34,35]	ν_s_ Al-O of [AlO_4_] [34,35]	Al-O of [AlO_6_] [34,35]	Complex AlO_4_ and AlO_6_ interactive Vibration [34]	ν_as_ (HO)-[Al]O [33]	δ Al-OH/ (O)H…O-H [36,37]	δ O-H [33]
S1	Position (cm^−1^)	484	541	631	754	888	937	1060	1113	1147
	Integral intensity	4.74	28.04	42.11	39.23	43.93	2.92	6.59	0.45	5.23
	Width (cm^−1^)	79	125	145	158	157	62	91	54	115
S2	Position (cm^−1^)	501	555	641	754	880	949	1068	1115	1170
	Integral intensity	4.08	16.12	25.16	27.98	32.6	5.2	3.76	1.27	3.22
	Width (cm^−1^)	92	133	159	166	173	92	95	78	106
S3	Position (cm^−1^)	501	551	637	752	885	946	1066	1114	1168
	Integral intensity	4.27	18.02	28.22	33.78	36.72	3.51	4.79	0.92	5.08
	Width (cm^−1^)	96	136	156	165	168	79	101	75	129

**Table 5:** Brunauer–Emmett–Teller (BET) measurements.

Sample Name	BET Surface Area (m^2^/g)	Pore Volume (cm^3^/g)
S1	238.395 ± 1.301	0.259
S2	274.524 ± 5.861	0.206
S3	271.328 ± 5.869	0.183

## Data Availability

The data presented in this study are available on request from the corresponding author.
